# The Impact of Combretastatin A-4 on Cancer Cells and Circulating Tumor Cells (CTCs): A Multi-Assay Approach

**DOI:** 10.32604/or.2026.085665

**Published:** 2026-09-14

**Authors:** Dimitrios Papakonstantinou, Vasileios Vardas, Despoina M. Varouhaki, Aikaterini Kotzamouratoglou, Karolina Mangani, Julia A. Ju, Catherine Alix-Panabières, Stuart S. Martin, Constantinos M. Athanassopoulos, Galatea Kallergi

**Affiliations:** 1 Laboratory of Biochemistry/Metastatic Signaling, Section of Genetics, Cell Biology and Development, Department of Biology, University of Patras, University Campus, Patras, Greece; 2 Synthetic Organic Chemistry Laboratory, Department of Chemistry, University of Patras, University Campus, Patras, Greece; 3 Department of Pharmacology and Physiology, University of Maryland School of Medicine, Baltimore, MD, USA; 4 Laboratory of Rare Human Circulating Cells and Liquid Biopsy (LCCRH), University Medical Centre of Montpellier, Montpellier, France; 5 CREEC, MIVEGEC, University of Montpellier, CNRS, IRD, Montpellier, France; 6 European Liquid Biopsy Society (ELBS), Hamburg, Germany

**Keywords:** Combretastatin A-4, circulating tumor cells, microtubules, microtentacles, metastasis

## Abstract

**Objectives:** Combretastatin A-4 (CA-4) is a microtubule-disrupting agent with established anti-tumor properties. This study aimed to evaluate the effects of CA-4 on key metastatic traits of cancer cells, including migration, clonogenic potential, cytoskeletal protein expression, and microtentacle (McTN) formation, using multiple cancer cell models, including the colon patient-derived circulating tumor cell line CTC-MCC-41. **Methods:** H1299 (non-small cell lung cancer), MDA-MB-231 (triple-negative breast cancer), HT-29 (colorectal cancer), and CTC-MCC-41 (derived from the blood of a colon cancer patient) cells were treated with CA-4 (10 μM) for 24 and 48 h. Colony formation was assessed with a clonogenic assay. Migration assays (Boyden chamber) evaluated cell motility, while cell viability was determined via MTT assay. Western blot analysis examined vimentin and α/β-tubulin expression. TetherChip assay analyzed McTN formation using wheat germ agglutinin (WGA) staining. **Results:** CA-4 treatment significantly reduced cell viability, colony formation, and migration across all tested cancer cell lines. Western blot analysis revealed a marked reduction in vimentin and α/β-tubulin expression after 48 h of treatment, indicating disruption of cytoskeletal integrity. In addition, TetherChip analysis demonstrated a pronounced decrease in McTN formation following CA-4 exposure, suggesting inhibition of cytoskeletal protrusions associated with metastatic dissemination. **Conclusions:** CA-4 effectively impairs multiple cancer cell functions related to metastatic progression, including proliferation, migration, cytoskeletal organization, and McTN formation. These findings highlight the potential of CA-4 as a microtubule-targeting agent with anti-metastatic activity, particularly in circulating tumor cells.

## 1 Introduction

Microtubules are essential components of the cytoskeleton, and play key roles in numerous cellular processes, including mitotic spindle formation, intracellular transport, and maintenance of cellular architecture [1,2,3]. Due to their central role in cell division, microtubules have long been targeted in cancer therapy through agents that either stabilize or depolymerize them, leading to mitotic arrest and apoptosis [4,5]. Taxanes (e.g., paclitaxel, docetaxel) and vinca alkaloids (e.g., vinblastine, vincristine) are well-established microtubule-targeting agents (MTAs) used in clinical practice. However, resistance frequently develops during treatment, increasing the need for compounds that target microtubules through distinct mechanisms of action [6,7,8]. Furthermore, taxanes have been reported to potentially promote metastatic spread, highlighting the need for new approaches that avoid microtubule stabilization [9,10].

Combretastatin A-4 (CA-4) is a vascular-disrupting compound that binds to the colchicine-binding site of tubulin, leading to microtubule depolymerization and disruption of cellular architecture [11]. It has been shown to induce mitotic arrest, trigger apoptosis, and impair tumor vasculature, thereby limiting the supply of oxygen and nutrients to tumor cells [12,13,14]. While its cytotoxic and anti-angiogenic properties have been extensively investigated, its effects on metastatic traits such as cell motility, adhesion, and cytoskeletal remodeling remain insufficiently explored. Given the critical role of microtubule stability in maintaining cell shape and enabling metastatic progression [15], additional studies examining the effects of CA-4 on cytoskeletal organization remain necessary.

Microtentacles (McTNs), tubulin-rich membrane protrusions that extend from the cell surface [16,17], have emerged as an important cytoskeletal structure associated with metastatic progression. Evidence suggests that McTNs participate in several stages of the metastatic cascade, by promoting tumor cell detachment from the primary tumor, survival in circulation, and reattachment to secondary sites [18]. McTNs are particularly relevant in the context of circulating tumor cells (CTCs), which detach from the primary tumor and enter the bloodstream, actively acting as mediators of metastatic dissemination. By promoting interactions between tumor cells and surrounding blood components, McTNs may enhance CTC survival in the circulation, thereby facilitating colonization of distant tissues [19,20,21,22,23]. Since McTNs rely on microtubule integrity, targeting their formation through microtubule-disrupting agents represents a compelling strategy to impair key mechanisms of metastasis.

Considering the importance of microtubules in both cell division and McTN formation, we investigated whether CA-4 could impair metastatic behavior by disrupting multiple tumor-associated functions. Therefore, the aim of the present study was to comprehensively evaluate the effects of CA-4 on cell via-bility, clonogenic potential, migration, cytoskeletal protein expression, and McTN formation in repre-sentative models of breast, non-small cell lung cancer (NSCLC), and colorectal cancer. In addition, we assessed its effects in the first patient-derived colon cancer CTC cell line, CTC-MCC-41 [24], to better reflect the biology of metastatic disease and to provide insight into the potential of CA-4 as an an-ti-metastatic therapeutic strategy targeting CTCs.

## 2 Materials and Methods

### 2.1 Synthesis and Characterization of CA-4

CA-4 was synthesized from commercially available starting materials according to previously reported methods [25,26,27]. The synthesized compound was confirmed to be chemically identical to natural (Z)-Combretastatin A-4 (cis-form) based on spectroscopic and analytical characterization. Detailed characterization data, including nuclear magnetic resonance (NMR) spectra, mass spectrometry (MS) data, melting point, and high-performance liquid chromatography (HPLC) purity, are provided in Supplementary Section S1 (Supplementary Figs. S1–S10) and Supplementary Table S1.

### 2.2 Cell Culture

The human cancer cell lines H1299 (CRL-5803, RRID: CVCL_0060), MDA-MB-231 (HTB-26, RRID: CVCL_0062), and HT-29 (HTB-38, RRID: CVCL_0320) were maintained in Dulbecco’s Modified Eagle Medium (DMEM) GlutaMAX (Thermo Fisher Scientific, 10566016, Waltham, MA, USA) supplemented with 10% fetal bovine serum (FBS, PAN-Biotech, P40-37500, Aidenbach, Germany) and 50 U/mL penicillin/50 μg/mL streptomycin (15140122, Thermo Fisher Scientific) (defined as full medium). The CTC-MCC-41 cell line (RRID: CVCL_0I26), a patient-derived colon cancer CTC model [24], was cultured in Roswell Park Memorial Institute 1640 medium (RPMI 1640, 21875034, Thermo Fisher Scientific) supplemented with 10% FBS (P40-37500, PAN-Biotech), and 1% insulin-transferrin-selenium (51300044, Thermo Fisher Scientific, MA, USA), epidermal growth factor (EGF; 20 ng/mL) (Qkine, QK011-0100, Cambridge, UK), and fibroblast growth factor (FGF; 10 ng/mL) (QK025-0050, Qkine) (defined as full medium). CTC-MCC-41 cells were grown in ultra-low attachment flasks (Corning, 3814, NY, USA). Cells were maintained at 37°C in 5% CO_2_. Cells were passaged using 0.25% trypsin-EDTA (25200056, Thermo Fisher Scientific). The patient-derived colon cancer CTC-MCC-41 cell line was kindly provided by Prof. Alix-Panabières (University Medical Centre of Montpellier, France). All other cancer cell lines were obtained from the American Type Culture Collection (ATCC) (Manassas, VA, USA).

The cell lines were selected to give a broader aspect of CA-4’s effects across different cancer types with distinct characteristics. H1299 (NSCLC) and MDA-MB-231 (TNBC) are representative models of highly metastatic tumor types. CTC-MCC-41 cells, derived from a colorectal cancer patient, were included because they represent a circulating tumor cell model. HT-29 cells are an established colorectal cancer cell line, allowing assessment in an additional epithelial cancer type corresponding to the colorectal cancer tissue. All cell lines were authenticated using short tandem repeat (STR) profiling, and all experiments were performed with mycoplasma-free cells.

### 2.3 Cell Viability (MTT)

A dose-response analysis was performed in H1299 cells to determine the IC_50_ value of CA-4 after 24 h of treatment (Fig. S11). The concentration used in all subsequent experiments was selected based on this analysis and previous studies evaluating the effects of CA-4 in cancer cell models [28,29,30,31].

Cells were seeded in 48-well plates at a density of 15,000 cells per well for adherent cell lines (H1299, MDA-MB-231, HT-29) and 20,000 cells per well for the CTC-MCC-41 line. Cells were initially cultured in full medium for 24 h and subsequently serum-starved for an additional 18 h. This serum starvation condition was performed to minimize the influence of serum-derived growth factors and to establish consistent baseline conditions before CA-4 treatment, minimizing starvation-related cytotoxicity in different conditions/experiments (adherent, non-adherent, TetherChip, etc.).

Following serum starvation, the medium was replaced with fresh serum-free medium containing either CA-4 (10 μM) or dimethyl sulfoxide (DMSO) (control, final concentration 0.1% v/v) and cells were incubated for 24 or 48 h. Cell viability was assessed using the 3-(4,5-dimethylthiazol-2-yl)-2,5-diphenyltetrazolium bromide (MTT) assay. Briefly, 1/10 of the medium’s volume of MTT [5 mg/mL in phosphate-buffered saline (PBS, Zeus Scientific, FA0008S, Branchburg, NJ, USA)] was added to each well, resulting in a final concentration of 0.5 mg/mL. Plates were incubated for 2 h at 37°C, protected from light. Formazan crystals were solubilized with acidified isopropanol (PanReac AppliChem ITW Reagents, 131090, Monza, Italy) and the absorbance was measured at 550 nm using a MRX microplate reader (Dynex Technologies, Chantilly, VA, USA). Background absorbance was corrected by subtracting the mean optical density (OD) of cell-free (culture medium-containing) wells from all experimental readings.


$$Corrected_{OD}=OD_{sample}-OD_{blank}$$

**Cell viability was calculated using the following formula:**


$$Cell\;viability\;\left(\%\right)=\frac{Corrected\;OD_{treated}}{Corrected\;OD_{control}}\times 100$$

**Experiments were performed in three independent biological replicates.**

### 2.4 Clonogenic Assay

The clonogenic assay was performed to assess the long-term proliferative capacity of tumor cells through colony formation. This assay was performed on both adherent (H1299, MDA-MB-231, HT-29) and non-adherent (CTC-MCC-41) cancer cells following CA-4 treatment for 24 or 48 h.

For adherent cell lines (H1299, MDA-MB-231, HT-29), cells were seeded at 1000 cells per well in six-well plates and allowed to adhere for 2 days in a 5% CO_2_ incubator. Cells were then serum-starved and treated with 10 μM CA-4, for 24 and 48 h, and DMSO (0.1% v/v) in the treated and control groups, respectively. Following treatment, the medium was replaced with fresh complete medium, and cells were incubated for an additional 5 days to allow colony formation. Finally, the medium was gently removed, and cells were washed twice with PBS before adding 70% ethanol (793175, Sigma-Aldrich, St. Louis, MO, USA). After fixation for 15 min, ethanol was removed, and cells were air-dried for 30 min at room temperature (RT). Crystal violet (C0775, Sigma-Aldrich) solution (0.5% w/v) was then added and cells were incubated for 15 min at RT. Excess stain was gently rinsed with distilled water (dH_2_O), and plates were left inverted on tissue paper to dry overnight. Colonies consisting of at least 40 cells were counted manually using an Oxion Inverso inverted microscope (Euromex, Arnhem, The Netherlands). Plates were imaged using the Gel Doc XR+ Gel Documentation system (Bio-Rad Laboratories, Hercules, CA, USA).

CTC-MCC-41 cells (non-adherent cell line) were seeded at 2000 cells per well in ultra-low attachment six-well plates (Corning, 3471, NY, USA). Cells were cultured in full medium for 2 days before treatment and were subsequently serum-starved and treated with 10 μM CA-4 and DMSO (0.1% v/v) in the treated and control groups, respectively (24 or 48 h). After treatment, fresh full medium was added, and cells were incubated for 5 days, to allow colony formation. Colonies containing at least 40 cells were manually counted and imaged using an Oxion Inverso inverted microscope (Euromex). Colony counting was not performed in a blinded manner.

Experiments were performed in three independent biological replicates.

### 2.5 Migration Assay

Transwell assays were used to evaluate the migratory capacity of H1299, MDA-MB-231, HT-29, and CTC-MCC-41 cells following treatment with CA-4. ThinCert™ cell culture inserts (3-μm pore size) (662630, Greiner Bio-One, Monroe, USA) were placed into a 24-well plate, and 100 μL of plain medium was added to each insert to hydrate the membrane. After the medium had passed to the lower chamber (~1–2 min), 750 μL of full medium containing CA-4 (10 μM) or DMSO (control, 0.1% v/v) was added to the lower chamber. Cells were counted, and 1 × 10^5^ cells per well were resuspended in 200 μL of serum-free medium without CA-4 and seeded into the upper chamber of each insert. CA-4 treatment was applied exclusively through the lower chamber. This setup was used to maintain the serum-driven chemoattractant gradient required for cell migration. Plates were incubated at 37°C with 5% CO_2_ for 24 and 48 h to allow migration.

Following incubation, inserts were carefully transferred to a new 24-well plate for processing. Non-migrated cells from the upper side of the insert membrane were removed using a cotton swab. Inserts were then fixed with 3.7% formaldehyde for 2 min, followed by incubation with 100% methanol (ME0302, Scharlau, Barcelona, Spain) for 20 min at RT. Cells that had migrated to the lower surface of the membrane were stained with 0.05% (w/v) crystal violet (C0775, Sigma-Aldrich) for 20 min at RT, protected from light. Excess stain was removed by washing twice with PBS.

Inserts were transferred onto glass slides and examined for imaging using an Oxion Inverso inverted microscope (Euromex). For quantification, 120 μL of 100% methanol was added to each insert followed by incubation for 15 min at RT. The solution was then centrifuged at 10,000× *g* for 1 min, and the supernatant was transferred to a 96-well plate for absorbance measurement at 550 nm using a MRX microplate reader (Dynex Technologies). Experiments were performed in three independent biological replicates.

### 2.6 Western Blot Analysis

Cells were routinely passaged and seeded into three separate culture flasks per cell line. After allowing the cells to reach 70–80% confluency, cells were then serum-starved and treated with DMSO (vehicle control, 0.1% v/v) and 10 μM CA-4 for 24 or 48 h. Protein lysates were prepared from H1299, MDA-MB-231, HT-29, and CTC-MCC-41 cells following CA-4 treatment. Cells were washed three times with ice-cold PBS and lysed using lysis buffer (50 mM Tris pH 7.4, 150 mM NaCl, 1 mM EDTA, 1% Triton X-100, 0.1% SDS) supplemented with protease inhibitors (P8340, Sigma-Aldrich). Lysates were incubated on ice for 10 min, mechanically scraped, and centrifuged at 20,000× *g* for 30 min at 4°C to remove debris. Supernatants were collected and stored at −20°C until further use. Protein concentration was quantified using the Bradford assay.

Equal amounts of protein (20 μg) were separated by SDS-PAGE on 10% polyacrylamide gels and transferred onto Immobilon-P PVDF membranes (IPVH00010, Merck KGaA, St. Louis, MO, USA) using the Trans-Blot Turbo Transfer System (1704150, Bio-Rad, USA) (25 V, 1.0 A, 45 min). Membranes were blocked with 5% bovine serum albumin (BSA) (A2244, AppliChem GmbH, Darmstadt, Germany) in Tris-buffered saline (A2264, AppliChem) containing 0.05% Tween-20 (A4974, AppliChem) (TBS-T) for 1 h at RT. Membranes were then incubated overnight at 4°C with the following primary antibodies diluted in 5% BSA: mouse anti-vimentin (1:1000) (sc-6260, Santa Cruz Biotechnology, Dallas, TX, USA), rabbit anti-α/β-tubulin (1:1000) (2148S, Cell Signaling Technology, Danvers, MA, USA), and mouse anti-actin (1:1000) (sc-8432, Santa Cruz Biotechnology), which was used as a loading control. Membranes were then incubated for 1 h at RT with horseradish peroxidase (HRP)-conjugated secondary antibodies: goat anti-mouse IgG-HRP (1:2500) (AP124P, Merck Millipore KGaA) for vimentin and actin, and goat anti-rabbit IgG-HRP (1:2500) (AP132P, Merck KGaA) for α/β-tubulin. Before reprobing with a second primary antibody from the same host species, membranes were incubated in 0.5% sodium azide (NaN3, S2002, Sigma-Aldrich) in in TBS-Tween for 1 h at RT, followed by washing with TBS-T.

Protein bands were detected using SuperSignal™ West Pico PLUS Chemiluminescent Substrate (34580, Thermo Fisher Scientific) according to the manufacturer’s instructions. Band intensities were quantified using the ImageJ software (version 1.54p, National Institutes of Health, Bethesda, MD, USA).

Experiments were performed in three independent biological replicates.

### 2.7 TetherChip Analysis

Cancer cell lines (H1299, MDA-MB-231, HT-29, and CTC-MCC-41) were cultured for 4 days in 6-well plates (30006, SPL Life Sciences, Pocheon-si, South Korea) pre-coated with 10 mg/mL poly(2-hydroxyethyl methacrylate) (polyHEMA; 529257-1G, Sigma-Aldrich), as previously described [22]. Cell culture conditions were maintained as previously described [22], with medium renewal occurring every two days. Prior to CA-4 treatment, cells were maintained in serum-free medium for 18 h to deplete serum factors. Cells were subsequently exposed to 10 μM CA-4 in serum-free medium for 1 h. DMSO was used as vehicle control, at a final concentration of 0.1% v/v. Following treatment, cells were seeded onto TetherChip wells at a density of 100,000 cells per well and allowed to tether for 45 min, as described in [21,23].

### 2.8 Immunofluorescence on TetherChips

Following tethering, immunofluorescence (IF) staining with wheat germ agglutinin (WGA; W11261, Thermo Fisher Scientific) was performed to assess McTN formation, as described in [22]. Fixation was carried out using 3.7% formaldehyde (252931, AppliChem) in PBS for 10 min at RT, followed by permeabilization with 0.1% Triton X-100 (HFH10, Thermo Fisher Scientific) in PBS for 10 min at RT. To reduce non-specific binding, cells were incubated in 5% FBS in PBS for 1 h at RT. To visualize McTNs, WGA conjugated to Alexa Fluor 488 (1:100 dilution in PBS/1% FBS; W11261, Thermo Fisher Scientific) was used, as WGA binds to mammalian cell membranes, enabling visualization of McTNs [32]. For nuclear staining, Hoechst nuclear stain (1:2500 dilution in PBS/1% FBS; 4082S, Cell Signaling Technology) was used. Stained cells were visualized using the VyCAP Puncher system version 1.5.1 (VyCAP B.V., Enschede, The Netherlands).

The entire TetherChip well was examined for each sample. Cells exhibiting membrane protrusions morphologically consistent with McTNs were classified as McTN-positive.

### 2.9 Statistical Analysis

Experimental data were normalized to the corresponding control group prior to statistical analysis. Protein expression levels were quantified by densitometric analysis using ImageJ software (version 1.54p, National Institutes of Health) [33] (National Institutes of Health, Bethesda, MD, USA) and normalized against actin, with control values set to 1. Data were analyzed using one-way ANOVA followed by Dunnett’s multiple comparisons test, comparing each treatment group to its respective untreated control. Results are expressed as mean ± SD. Statistical analyses were performed using GraphPad Prism (Version 8.0.1, GraphPad Software, San Diego, CA, USA). Statistical significance was defined as *p* < 0.05.

## 3 Results

### 3.1 CA-4 Reduces Cancer Cell Viability

To evaluate the cytotoxic effects of CA-4, MTT assays were performed in all four cancer cell lines, H1299, HT-29, MDA-MB-231, and CTC-MCC-41, following treatment with 10 μM CA-4 for 24 and 48 h. Cell viability was determined by measuring OD values, normalized to DMSO-treated control cells (set at 100%). Viability values for the treated samples were normalized to their corresponding controls; therefore, the control group is represented as 100% viability. Results were expressed as mean ± SD from three biological replicates. The estimated IC_50_ value of CA-4 in H1299 cells after 24 h treatment was 10.38 μM (Fig. S11).

**Figure 1:**
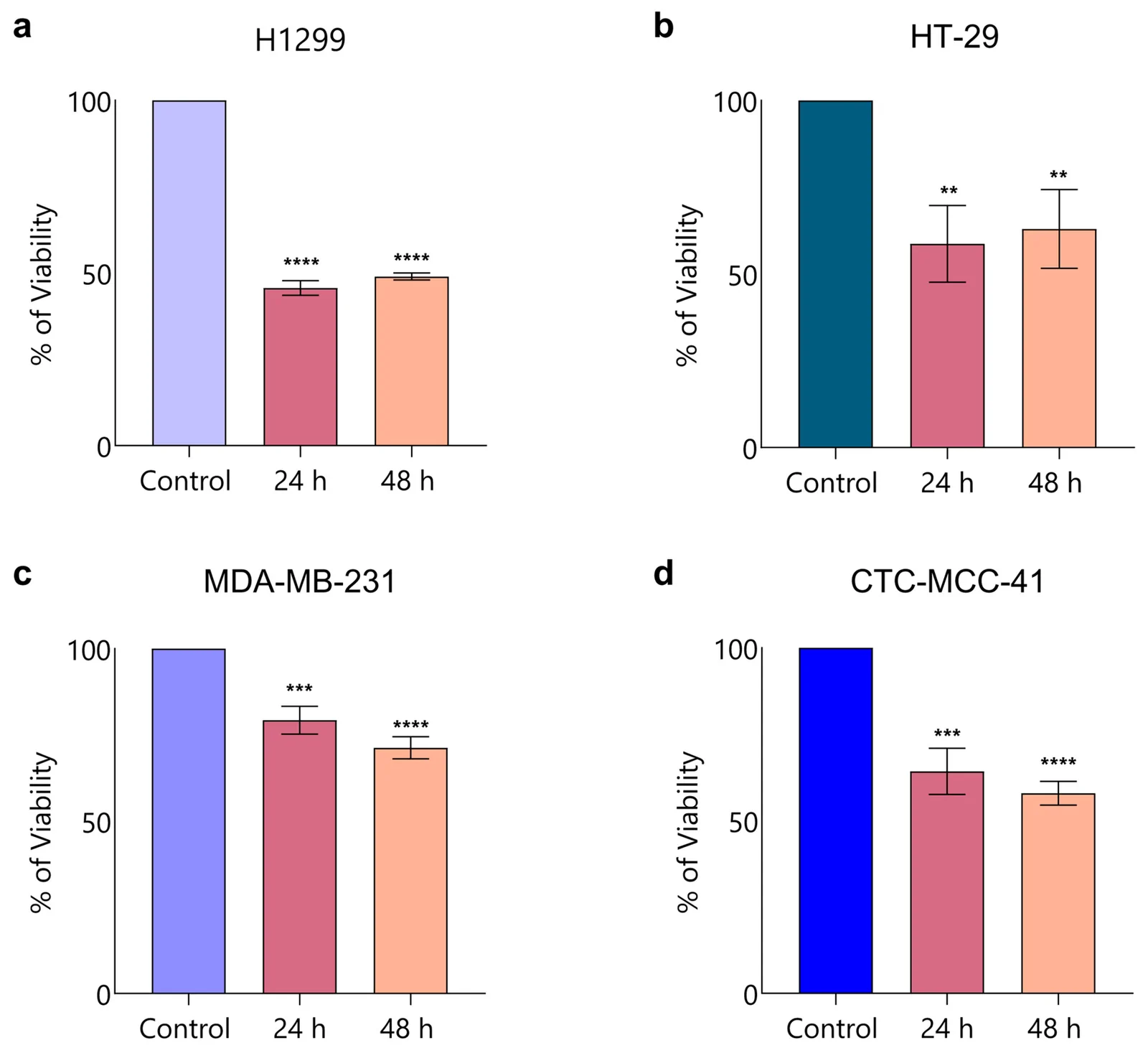
Combretastatin A-4 (CA-4) reduces cell viability in cancer cell lines. (**a**–**d**). Percentage of viability of H1299 (**a**), HT-29 (**b**), MDA-MB-231 (**c**), and CTC-MCC-41 (**d**) cells following treatment with CA-4 (10 μM) for 24 and 48 h. Cell viability was assessed using the MTT assay and is expressed as a percentage relative to untreated controls. Error bars represent the mean ± Standard Deviation (SD) from three independent experiments. Statistical significance was determined using an unpaired two-tailed *t*-test, with significance indicated as follows: **p ≤ 0.01; ***p ≤ 0.001; ****p ≤ 0.0001.

Exposure to CA-4 reduced viability in all four cell lines, although the magnitude of the response differed among models. In H1299 cells, viability decreased to 45.7% ± 2.1 after 24 h and remained at a similar level after 48 h treatment (49.0% ± 1.0; *p* < 0.0001 for both time points) (Fig. 1a), indicating a sustained cytotoxic effect. HT-29 cells also showed a marked reduction in viability, decreasing to 58.7% ± 11.1 at 24 h and 63.0% ± 11.4 at 48 h (*p* = 0.0027 and *p* = 0.0046, respectively) (Fig. 1b).

MDA-MB-231 cells exhibited a more moderate response, with viability reduced to 79.3% ± 4.0 at 24 h and further to 71.3% ± 3.2 after 48 h treatment (*p* = 0.0003 and *p* < 0.0001, respectively) (Fig. 1c). In CTC-MCC-41 cells, viability decreased to 64.3% ± 6.7 at 24 h (*p* = 0.0001) and further to 58.0% ± 3.5 after 48 h (*p* < 0.0001) (Fig. 1d), demonstrating a time-dependent response to CA-4 treatment.

H1299 cells displayed the greatest inhibition of viability, whereas MDA-MB-231 cells appeared comparatively less sensitive to treatment. Collectively, these results indicate a significant cytotoxic effect of CA-4 across multiple cancer cell models, including the patient-derived CTC-MCC-41 line.

### 3.2 CA-4 Treatment Impairs Colony Formation

**Figure 2:**
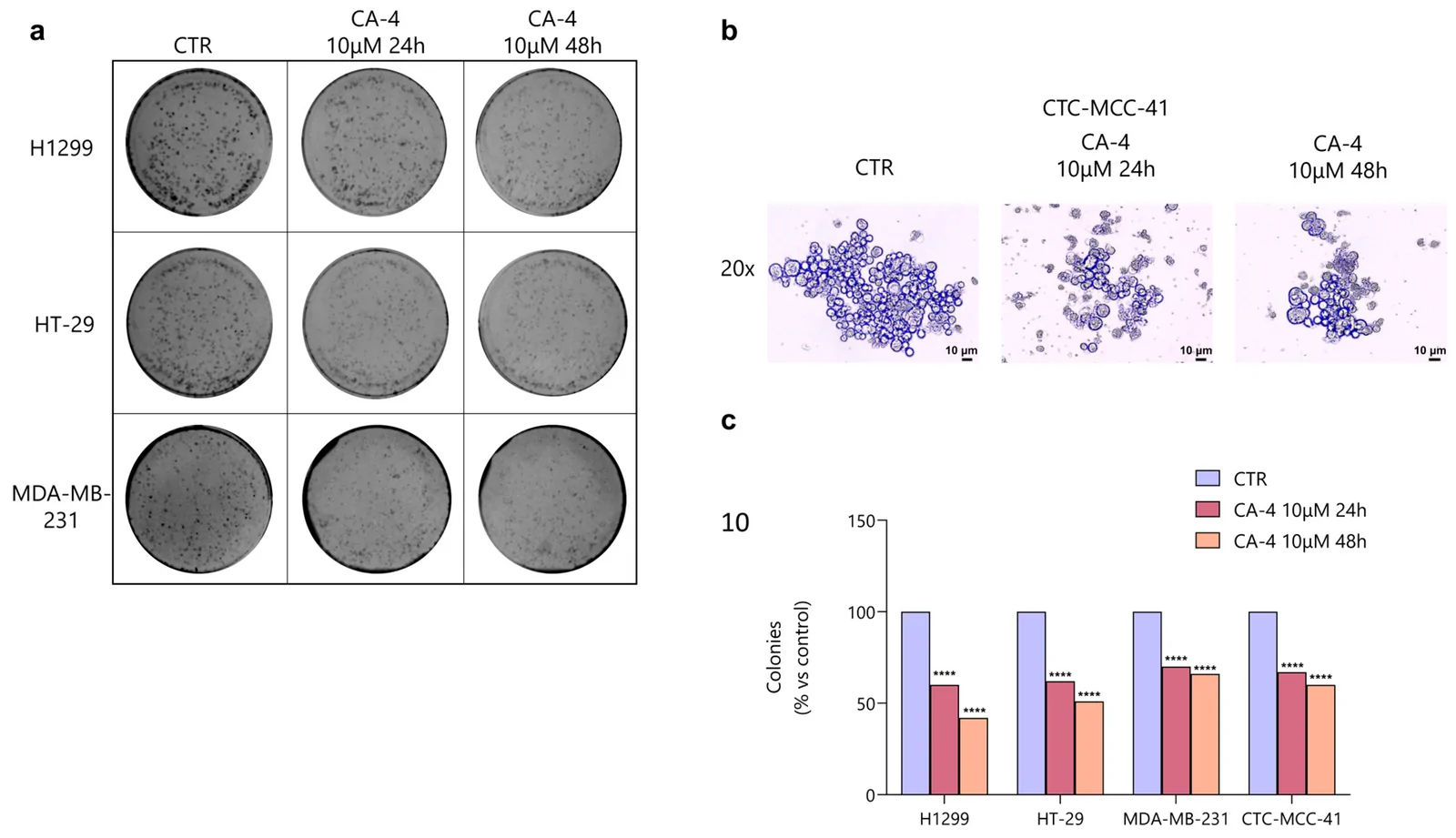
CA-4 treatment impairs colony formation ability in cancer cell lines. (**a**). Representative plate-stained images of colony formation assays in H1299, HT-29, and MDA-MB-231 cells treated with CA-4 (10 μM) for 24 and 48 h. (**b**). Representative optical microscopy images showing colony morphology of CTC-MCC-41 cells under control and CA-4 treatment conditions (10 μM, 24 and 48 h), imaged at 10× and 20× magnifications. Scale bars represent 10 μm. (**c**). Quantification of colony formation shown as percentage relative to control (CTR). Data represent mean ± SD from three independent experiments. Statistical significance was determined using an unpaired two-tailed *t*-test, with significance indicated as follows: ****p ≤ 0.0001.

To evaluate the effects of CA-4 on clonogenic potential, colony formation assays were performed in three adherent cancer cell lines, H1299 (NSCLC), HT-29 (colorectal cancer), and MDA-MB-231 (TNBC), as well as in the first patient-derived colon cancer CTC cell line CTC-MCC-41 [24] (Fig. 2a,b), which was cultured in suspension. Crystal violet-stained colonies from the adherent cell lines are shown in Fig. 2a, while Fig. 2b depicts representative colonies from the non-adherent CTC-MCC-41 cell line. Microscope images are provided in Supplementary Fig. S12.

Cells were treated with 10 μM CA-4 for 24 h and 48 h. Colony counts were normalized to DMSO-treated controls (100%) and are presented as mean ± SD from three biological replicates.

CA-4 exposure markedly impaired colony-forming ability in all tested cell lines in a time-dependent manner (Fig. 2c), with colony formation values expressed as percentages relative to the corresponding control group. After 24 h treatment, colony numbers decreased, relative to controls, by 40% in H1299 cells (control: 196.0 ± 4.4 vs. treated: 117.3 ± 2.1 colonies, *p* < 0.0001), 38% in HT-29 cells (185.0 ± 11.4 vs. 114.7 ± 5.5, *p* < 0.0001), 32% in MDA-MB-231 cells (116.0 ± 7.6 vs. 79.3 ± 4.9, *p* < 0.001), and 33% in CTC-MCC-41 cells (55.0 ± 8.1 vs. 37.0 ± 2.6, *p* < 0.0001) relative to controls.

A stronger suppression of colony formation was observed after 48 h of treatment (Fig. 2c). H1299 colonies were reduced by 58% compared to control levels (82.3 ± 4.0, *p* < 0.0001), while HT-29 and CTC-MCC-41 cells showed reductions of 49% (90.7 ± 2.9, *p* < 0.0001) and 40% (32.7 ± 1.5, *p* < 0.0001), respectively. MDA-MB-231 cells also demonstrated a significant reduction in colony formation (34%; 78.0 ± 2.6, *p* < 0.001), although the effect was less pronounced compared to the other cell lines.

Across both treatment durations, H1299 (NSCLC) emerged as the most sensitive to CA-4 treatment, with the steepest decline in colony-forming capacity. The non-adherent CTC-MCC-41 model also exhibited marked sensitivity to treatment.

### 3.3 CA-4 Reduces Cell Motility

To evaluate the effects of CA-4 on cancer cell motility, Transwell migration assays were performed in H1299, HT-29, MDA-MB-231, and CTC-MCC-41 cells following treatment with 10 μM CA-4 for 24 h and 48 h. Migration was quantified by OD measurements normalized to untreated controls (set at 100%). Data are presented as mean ± SD from three biological replicates.

**Figure 3:**
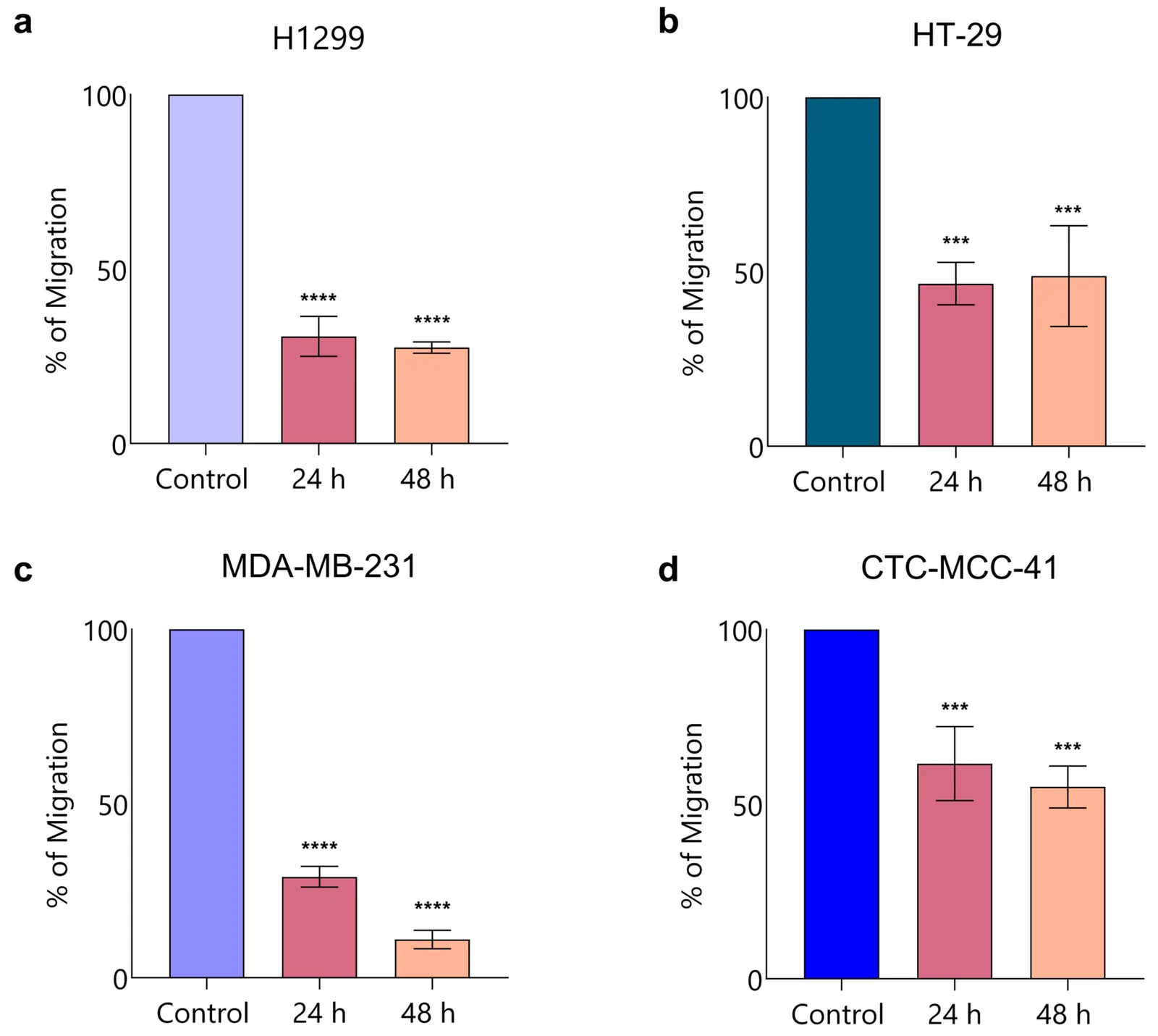
CA-4 treatment significantly impairs migration in cancer cell lines. (**a**–**d**). Quantification of cell migration in H1299 (**a**), HT-29 (**b**), MDA-MB-231 (**c**), and CTC-MCC-41 (**d**) cells treated with CA-4 (10 μM) for 24 and 48 h. Migration is expressed as a percentage relative to untreated control cells. Error bars represent the mean ± SD from three independent experiments. Statistical significance was determined using an unpaired two-tailed *t*-test, with significance indicated as follows: ***p ≤ 0.001; ****p ≤ 0.0001.

Migration was substantially reduced following CA-4 treatment in all tested cell lines, with the degree of inhibition varying across cell lines. In H1299 cells, migration decreased to 30.7% ± 5.7 after 24 h and 27.5% ± 1.6 after 48 h (*p* < 0.0001 for both time points), indicating a sustained inhibitory effect (Fig. 3a). HT-29 cells also exhibited significantly reduced migration, with values decreasing to 46.6% ± 6.1 at 24 h (*p* = 0.0007) and 48.8% ± 14.4 at 48 h (*p* = 0.0008) (Fig. 3b).

MDA-MB-231 cells, which are characterized by an aggressive and highly migratory phenotype [34], showed a particularly strong response to CA-4 treatment. Migration was reduced to 29.0% ± 3.0 after 24 h and further to 11.0% ± 2.6 after 48 h (*p* < 0.0001 for both) (Fig. 3c), representing the most pronounced inhibitory effect among the tested cell lines. In CTC-MCC-41 cells, migration decreased to 61.7% ± 6.1 after 24 h and further to 55.0% ± 5.0 after 48 h treatment (*p* = 0.0010 and *p* = 0.0004, respectively) (Fig. 3d), demonstrating that CA-4 effectively suppresses motility in both adherent and suspension cancer cell models.

Taken together, these results show that CA-4 effectively suppresses cancer cell migration across multiple tumor models, including the patient-derived CTC-MCC-41 line. Representative images of crystal violet-stained Transwell membranes are provided in Supplementary Fig. S13.

### 3.4 CA-4 Reduces Cytoskeletal Protein Expression

**Figure 4:**
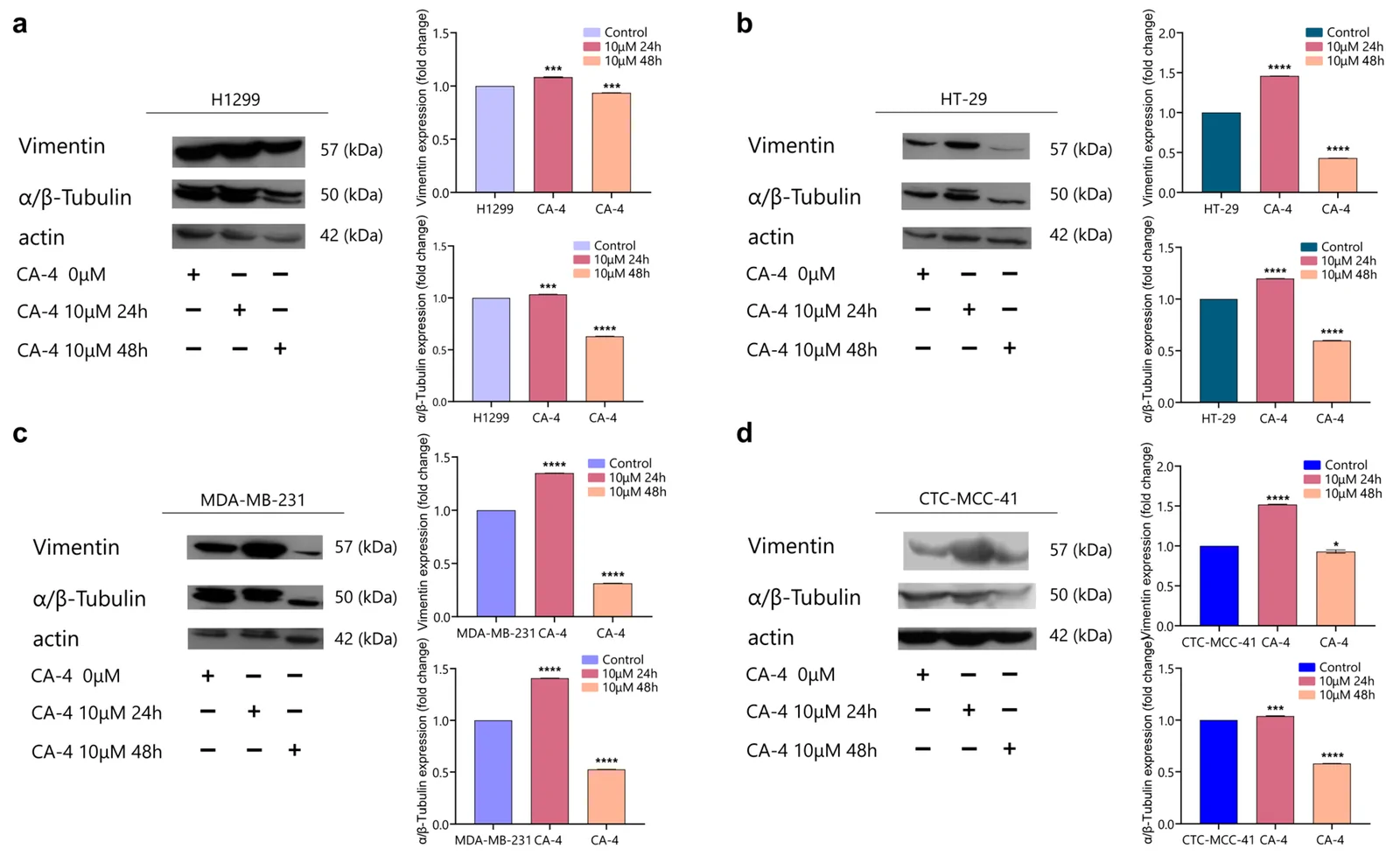
CA-4 disrupts cytoskeletal protein expression in cancer cell lines. (**a**–**d**). Western blot analysis of vimentin and α/β-tubulin levels in H1299 (**a**), HT-29 (**b**), MDA-MB-231 (**c**), and CTC-MCC-41 (**d**) cells following CA-4 treatment (10 μM) for 24 and 48 h. actin was used as a loading control. Densitometric quantification of vimentin and α/β-tubulin is shown as fold change relative to untreated control. Error bars represent the mean ± SD from three independent experiments. Statistical significance was determined using an unpaired two-tailed *t*-test, with significance indicated as follows: *p ≤ 0.05; ***p ≤ 0.001; ****p ≤ 0.0001.

To evaluate the effects of CA-4 on cytoskeletal protein expression, Western blot analysis was performed in H1299, HT-29, MDA-MB-231, and CTC-MCC-41 cells following treatment with 10 μM CA-4 for 24 h and 48 h. Protein expression levels of vimentin and α/β-tubulin were normalized against actin and quantified by densitometric analysis using ImageJ. Results are presented as percentage changes relative to DMSO-treated controls from three independent biological experiments. Representative Western blots are shown in Fig. 4a–d.

No reduction in vimentin expression was observed after 24 h of treatment. Instead, a moderate increase was observed in all tested cell lines, including H1299 (8.5%; *p* = 0.0002, Fig. 4a), HT-29 (46%; *p* < 0.0001, Fig. 4b), MDA-MB-231 (35%; *p* < 0.0001, Fig. 4c), and CTC-MCC-41 cells (52%; *p* < 0.0001, Fig. 4d). In contrast, prolonged exposure to CA-4 for 48 h resulted in a decrease in vimentin expression across all models, though the magnitude varied considerably between cell lines. Expression levels were reduced by 7% in H1299 cells (Fig. 4a; *p* = 0.0004), 57% in HT-29 cells (Fig. 4b; *p* < 0.0001), 69% in MDA-MB-231 cells (Fig. 4c; *p* < 0.0001), and 7.1% in CTC-MCC-41 cells (Fig. 4d; *p* = 0.016). The strongest reduction in vimentin expression was observed in the HT-29 and MDA-MB-231 models.

Comparable changes were observed for α/β-tubulin expression. After 24 h treatment, only minor increases were detected in H1299 (3%; *p* = 0.0002, Fig. 4a), HT-29 (20%; *p* < 0.0001, Fig. 4b), MDA-MB-231 (41%; *p* < 0.0001, Fig. 4c), and CTC-MCC-41 cells (4%; *p* = 0.0009, Fig. 4d). However, after 48 h treatment, α/β-tubulin levels were significantly reduced in all tested cell lines. Expression decreased by 36% in H1299 cells (Fig. 4a), 40% in HT-29 cells (Fig. 4b), 48% in MDA-MB-231 cells (Fig. 4c), and 42% in CTC-MCC-41 cells (Fig. 4d) (all *p* < 0.0001).

Collectively, prolonged CA-4 exposure resulted in marked reductions in α/β tubulin expression across all models, while the effect on vimentin was more variable, ranging from minimal (H1299, CTC-MCC-41) to pronounced (HT-29, MDA-MB-231), indicating cell-line-dependent alterations in cytoskeletal protein regulation.

### 3.5 CA-4 Suppresses Microtentacle Formation in Cancer Cells

**Figure 5:**
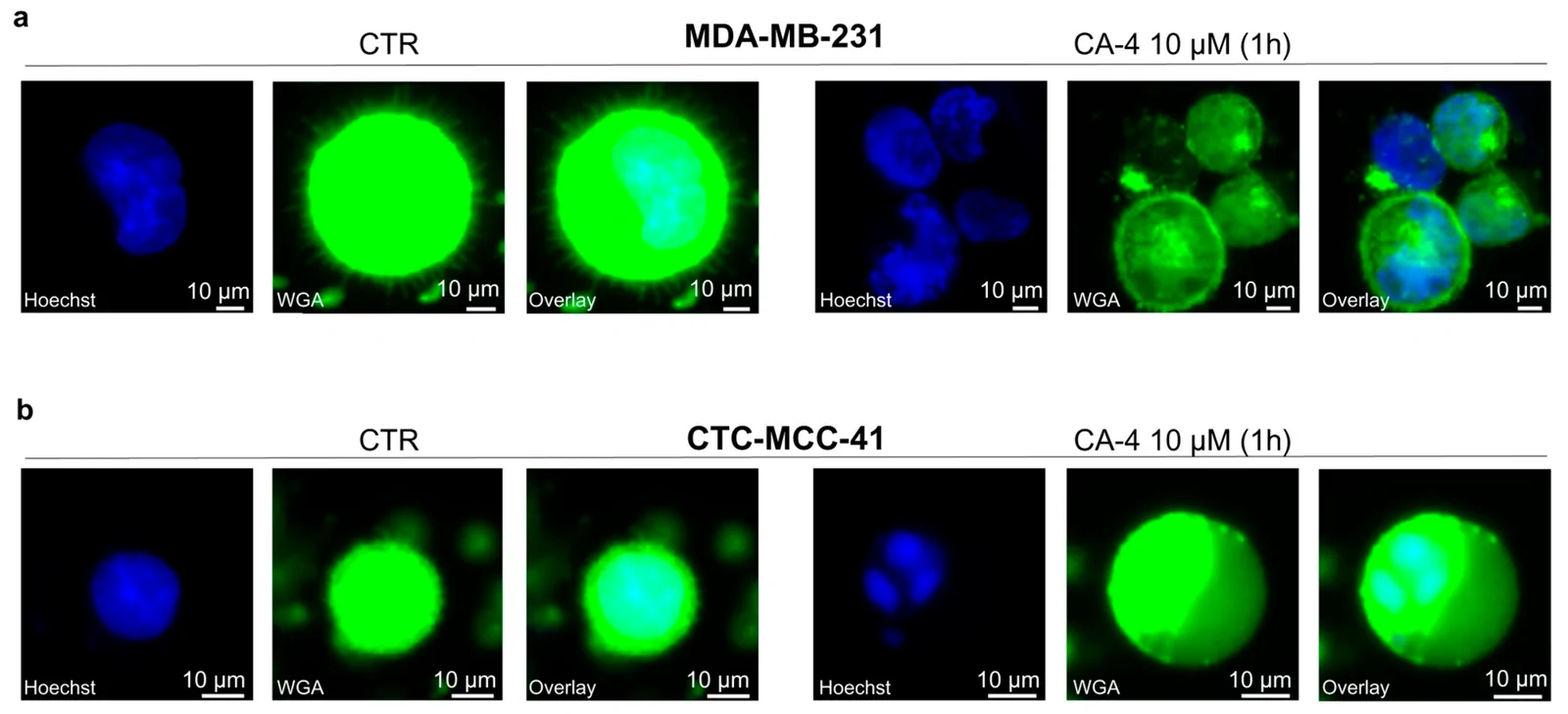
CA-4 treatment reduces microtentacle (McTN) formation in H1299 and HT-29 cells under non-adherent conditions. (**a**,**b**). Representative immunofluorescence images of H1299 (**a**) and HT-29 (**b**) cells seeded on TetherChip nanosurfaces and treated with CA-4 (10 μM) for 1 h. Cells were stained with Hoechst to visualize nuclei (blue) and WGA to label the plasma membrane (green). Image acquired from the VyCAP system (Magnification 40×). Scale bars represent 10 μm.

**Figure 6:**
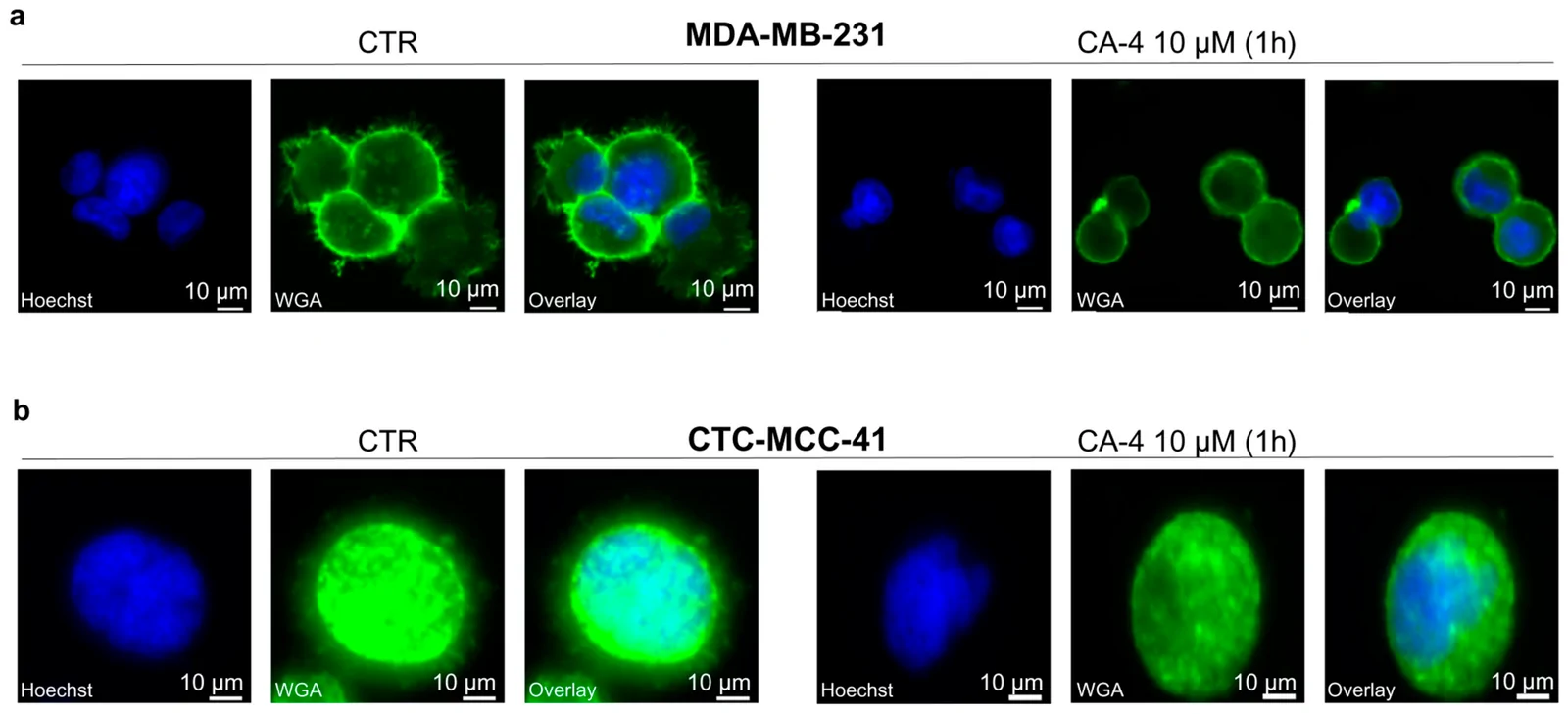
CA-4 treatment reduces microtentacle (McTN) formation in MDA-MB-231 and CTC-MCC-41 cells under non-adherent conditions. (**a**,**b**). Representative immunofluorescence images of MDA-MB-231 (**a**) and CTC-MCC-41 (**b**) cells seeded on TetherChip nanosurfaces and treated with CA-4 (10 μM) for 1 h. Cells were stained with Hoechst to visualize nuclei (blue) and WGA to label the plasma membrane (green). Image acquired from the VyCAP system (Magnification 40×). Scale bars represent 10 μm.

To assess the effect of CA-4 on microtentacle (McTN) formation, H1299, HT-29, MDA-MB-231, and CTC-MCC-41 cells were treated with 10 μM CA-4 for 1 h before immunofluorescence analysis. The shorter treatment duration was selected based on CA-4’s rapid effects on microtubule dynamics [35]. Representative fluorescence microscopy images of control and CA-4-treated cells are shown in Fig. 5 and Fig. 6. Cells were stained with WGA to visualize McTNs, and McTN-positive cells were quantified using the VyCAP platform. McTN-positive cells were manually counted in control and treated samples, and results were expressed as the percentage of McTN-positive cells relative to the corresponding untreated control group (set at 100%).

CA-4 treatment markedly reduced McTN formation in all tested cancer cell lines (Fig. S14).

In H1299 cells, McTN-positive cells decreased to 14.3% relative to control levels. HT-29 and MDA-MB-231 cells exhibited the greatest response, with values declining to 11.1% of their respective controls. Similarly, the patient-derived CTC-MCC-41 cell line showed a decrease to 31.4% of control levels following CA-4 treatment.

Notably, these effects were observed after only 1 h of treatment, indicating a rapid disruption of McTN formation following CA-4 exposure.

Together, these findings demonstrate that CA-4 rapidly suppresses McTN formation across multiple cancer cell models (Fig. S14), including the patient-derived CTC-MCC-41 line. Supplementary Table S2 summarizes the total number of McTN-positive cells identified across three technical replicate wells under control and treated conditions.

## 4 Discussion

Combretastatin A-4 is a microtubule-disrupting agent known for its potent cytotoxic and vascular-disrupting effects in cancer cells [36,37,38,39]. Although its ability to inhibit tubulin polymerization and interfere with mitotic progression has been extensively studied [37,38,39], its broader effects on metastatic traits associated with cytoskeletal remodeling remain to be fully elucidated [36,40,41]. In the present study, we investigated the effects of CA-4 across a panel of adherent cancer cell lines representing different solid tumors (H1299, HT-29, and MDA-MB-231), as well as the patient-derived metastatic CTC-MCC-41 model. Using complementary functional and molecular approaches, we evaluated whether CA-4 affects not only proliferation and viability, but also migration, cytoskeletal organization, and microtentacle formation.

MTT analysis demonstrated a significant reduction in viability across all adherent cancer cell lines following CA-4 treatment (Fig. 1a–c). The inhibitory effect was evident at 24 h and persisted after 48 h, indicating sustained cytotoxic activity. These findings are consistent with the established ability of CA-4 to disrupt microtubule dynamics and impair mitotic progression [42,43]. Similar cytotoxic responses have also been reported for vinorelbine, another microtubule-destabilizing agent that inhibits tubulin polymerization [44]. Vinorelbine (10 μM) significantly reduced viability in MDA-MB-231 and CTC-MCC-41 cells within 24 h [22], demonstrating a comparable time-dependent reduction in viability to that observed with CA-4 in the present study. Although CA-4 and vinorelbine target different tubulin-binding domains, both inhibit cancer cell proliferation through microtubule destabilization.

A time-dependent reduction in clonogenic capacity was observed across all tested cancer cell lines following CA-4 exposure (Fig. 2c). Colony formation progressively decreased between 24 and 48 h treatment, indicating sustained suppression of long-term proliferative capacity. Although the extent of inhibition varied among cell lines, all models demonstrated significant sensitivity to CA-4. Differences in responsiveness may reflect variations in proliferation rate, focal adhesion dynamics, and cytoskeletal organization between cancer cell types [45,46].

The anti-migratory activity of CA-4 was also evident across all adherent cancer cell models. Migration assays demonstrated a substantial reduction in cell motility following treatment, with stronger inhibition observed at 48 h compared to 24 h (Fig. 3). These findings suggest that CA-4 impairs migration in both well-differentiated and mesenchymal-like cancer cells, with MDA-MB-231 cells exhibiting the strongest response, potentially due to their increased dependence on dynamic cytoskeletal remodeling for migration [47].

The reduction in migratory capacity was accompanied by altered expression of the cytoskeletal proteins α/β-tubulin and vimentin, both of which play central roles in maintaining cellular architecture and motility. Western blot analysis demonstrated a significant downregulation of α/β-tubulin after 48 h treatment in all tested models (Fig. 4a–d). Vimentin expression followed a similar pronounced decrease in HT-29 and MDA-MB-231 cells, whereas the reduction was comparatively modest in H1299 and CTC-MCC-41 cells (Fig. 4a–d). Interestingly, a moderate increase in α/β-tubulin and vimentin expression was observed after 24 h treatment in all models. This transient upregulation may reflect an early adaptive response to cytoskeletal stress, as previously reported following exposure to microtubule-targeting agents [47,48,49].

Acute disruption of microtubule networks has been reported to trigger cytoskeletal remodeling responses that help cells maintain their structural integrity under stressful conditions [48,49]. One possible explanation for the transient increase in α/β-tubulin expression observed after 24 h of CA-4 treatment is the activation of such adaptive mechanisms. Tubulin levels are regulated through autoregulatory pathways that respond to changes in microtubule dynamics and integrity. Alterations in the balance between soluble and polymerized tubulin can affect tubulin mRNA stability, thereby modulating tubulin levels and contributing to the maintenance of microtubule homeostasis [50]. These autoregulatory mechanisms may help explain the initial increase in α/β-tubulin observed after 24 h, as cells attempt to compensate for CA-4-induced microtubule destabilization.

The increase in vimentin expression observed after 24 h may similarly reflect an early cellular response to CA-4-induced cytoskeletal stress. Vimentin intermediate filaments are functionally linked to the microtubule network and play important roles in maintaining cell architecture, polarity, migration, and mechanical stability [51,52,53]. In addition, vimentin has been implicated in cellular adaptation following mechanical or structural perturbations and contributes to the maintenance of cytoskeletal integrity under stress conditions [53,54]. The increase in vimentin expression observed after 24 h may be linked to the close functional interplay between intermediate filaments and microtubules during cytoskeletal remodeling. Given the role of vimentin in maintaining cellular architecture and mechanical stability, elevated vimentin levels may represent an early response to the structural perturbations induced by CA-4 treatment. However, both responses were transient, with sustained downregulation of both proteins evident by 48 h, most consistently for α/β-tubulin, and more variably for vimentin in a cell-line-dependent manner. (Fig. 4a–d). This finding suggests that sustained microtubule disruption ultimately overwhelms these compensatory mechanisms, though vimentin’s response to this appears to depend on intrinsic differences between the cell lines.

These molecular changes are particularly relevant because microtubules and intermediate filaments cooperate to regulate cell polarity, mechanical stability, and directional migration [55]. α/β-tubulin heterodimers form the microtubule network required for intracellular transport and force generation during cell movement, while vimentin contributes to cytoskeletal organization and mechanical support [47,51]. In addition, vimentin is strongly associated with epithelial-mesenchymal transition (EMT), a process linked to increased migration, invasion and metastatic potential [56,57]. Therefore, the simultaneous reduction of α/β-tubulin and vimentin expression following CA-4 treatment suggests that its anti-migratory effects extend beyond microtubule depolymerization alone and affect broader cytoskeletal interactions necessary for cancer cell motility.

Our findings are consistent with previous studies investigating the anti-metastatic properties of CA-4 and related compounds. Liang et al. [28] demonstrated that CA-4 inhibited proliferation, migration, and invasion in TPC1 thyroid cancer cells while simultaneously reducing vimentin expression through suppression of the PI3K/Akt signaling pathway. Similarly, Yang et al. [58] reported that the CA-4 analogue M410 disrupted microtubule organization and reduced HIF-1α expression in MDA-MB-231 cells, thereby impairing pro-metastatic signaling independently of mitotic arrest. Together, these findings support the notion that microtubule disruption can directly interfere with cellular mechanisms involved in metastatic progression.

In addition to impairing viability, clonogenicity, and migration, CA-4 markedly reduced McTN formation, a cytoskeletal feature associated with metastatic dissemination and CTC survival [59,60,61]. McTNs are tubulin-rich membrane protrusions that facilitate reattachment, aggregation, and interactions between tumor cells and components of the microenvironment [18,19,20]. To investigate this effect under non-adherent conditions, McTN formation was assessed using the TetherChip platform, which preserves suspended cell morphology and enables visualization of McTNs under circulation-like conditions [21,22,23,32].

CA-4 treatment significantly reduced McTN-positive cells across all tested cancer models after only 1 h of exposure (Fig. 5 and Fig. 6, Supplementary Fig. S14, Supplementary Table S2). The shorter treatment duration was selected based on previously established TetherChip protocols used for the evaluation of McTN-targeting agents [21,22,23]. McTNs are microtubule-dependent structures; therefore, disruption of microtubule polymerization can very rapidly alter their formation. Consistent with previous studies that employed short-term exposure (1 h), the marked reduction in McTN-positive cells observed after only 1 h of CA-4 treatment further supports the role of microtubule stability in the formation of these membrane protrusions. This rapid response highlights the sensitivity of McTNs to microtubule-targeting agents and further supports the role of microtubule integrity in maintaining these protrusive structures. The strongest inhibitory effect was observed in MDA-MB-231 cells, which also demonstrated the greatest reduction in migration, suggesting a strong dependence on cytoskeletal dynamics for metastatic behavior.

Recent studies further support the biological relevance of McTNs in metastasis. Ju et al. [23] demonstrated that tubulin-based McTNs promote heterotypic clustering between breast tumor cells and neutrophil-differentiated HL-60 cells, facilitating pro-metastatic interactions within the tumor microenvironment. In addition, Ju et al. [21] showed that McTNs contribute to tumor cell aggregation and reattachment under non-adherent conditions. Together, these findings suggest that suppression of McTN formation by CA-4 may represent an important mechanism through which microtubule-disrupting agents impair metastatic dissemination.

Particular emphasis was placed on the patient-derived CTC-MCC-41 model, as CTCs are considered key mediators of metastatic dissemination and are strongly associated with poor clinical outcomes [62,63]. However, the low abundance of CTCs in peripheral blood has limited their extensive characterization and the establishment of stable patient-derived CTC lines [24,64,65]. CTC-MCC-41, established by the group of Alix-Panabières, represents the first long-term stable colon cancer CTC line derived from a metastatic patient [24]. Given that metastatic cells often display biological and therapeutic characteristics distinct from those of primary tumor cells [66], this model is particularly suitable for investigating metastatic behavior and evaluating the therapeutic potential of anti-cancer agents.

In agreement with the findings observed in adherent cancer cell lines, CA-4 treatment significantly reduced viability, migration, colony formation, and McTN formation in CTC-MCC-41 cells. In addition, substantial downregulation of α/β-tubulin was observed after prolonged treatment, whereas the reduction in vimentin expression was comparatively modest (Fig. 4d). The reduction in clonogenicity may indicate impaired cluster-forming capacity, which is particularly relevant given the strong association between CTC clusters and metastatic potential [67]. By disrupting cytoskeletal organization, CA-4 may compromise the structural stability required for CTC aggregation, survival in circulation, and successful metastatic colonization. Similarly, the marked reduction in McTN-positive cells following treatment further supports the notion that CA-4 interferes with cytoskeletal mechanisms that facilitate CTC-mediated dissemination. Collectively, these findings highlight the potential of CA-4 to directly target metastatic properties of circulating tumor cells.

The examined cancer cell lines, derived from different tissue origins, exhibited differential sensitivity to CA-4 across the various functional assays. While H1299 cells exhibited the most pronounced reductions in cell viability and clonogenic capacity, MDA-MB-231 cells were particularly sensitive to the inhibitory effects of CA-4 on migration and McTN formation. Although the molecular basis of these differences was not specifically investigated in the present study, they likely reflect the distinct biological characteristics of each cell model. Cell lines from different cancer types vary in several features that can influence their response to microtubule-targeting agents, including their cytoskeletal organization, migratory potential, mechanical properties, and EMT status [48]. For example, MDA-MB-231 cells are known for their highly mesenchymal and invasive phenotype, which may explain why CA-4 had a particularly strong effect on migration and McTN formation in this cell model [68]. In contrast, H1299 cells appeared more susceptible to the effects of CA-4 on viability and colony formation, which may indicate a greater reliance on microtubule-mediated processes involved in proliferation and survival. Together, these observations indicate that the response to CA-4 is influenced by the biological context of each cancer cell type and may vary according to the cellular pathways that are most critical for maintaining its malignant phenotype.

The present study was performed using *in vitro* models, which provide a controlled environment for investigating the biological effects of CA-4 but cannot fully reproduce the complexity of metastatic disease. Nevertheless, the consistent findings across several cancer cell lines, including the patient-derived CTC-MCC-41 model, support the biological relevance of the observed effects. Additional studies in appropriate *in vivo* models will help to further evaluate the anti-metastatic potential of CA-4 and clarify the mechanisms underlying its activity.

Overall, the present study demonstrates that CA-4 exerts broad anti-cancer and anti-metastatic effects across multiple tumor models. Beyond its cytotoxic activity, CA-4 significantly impaired migration, clonogenicity, cytoskeletal protein expression, and McTN formation, indicating extensive disruption of cellular mechanisms associated with metastatic progression. Importantly, the pronounced effects observed in the patient-derived CTC-MCC-41 model further support the therapeutic relevance of CA-4 in targeting circulating tumor cells and metastasis-associated phenotypes.

## 5 Conclusions

This study demonstrates that CA-4 disrupts multiple cancer cell functions in both adherent tumor cell lines and the patient-derived CTC-MCC-41 model. CA-4 consistently reduced viability, colony formation, migration, and microtentacle formation, while also altering the expression of key cytoskeletal proteins involved in metastatic behavior. These findings confirm the cytotoxic activity of CA-4 and further support its role in impairing cytoskeletal mechanisms associated with cancer cell dissemination. Importantly, the pronounced effects observed in CTC-MCC-41 cells highlight the therapeutic relevance of CA-4 in circulating tumor cell-targeted approaches. Overall, our findings support further investigation of CA-4 as a microtubule-disrupting agent with potential anti-metastatic properties.

## Data Availability

The authors confirm that the data supporting the findings of this study are available within the article and its Supplementary Materials.

## Supplementary Materials

The supplementary material is available online at https://www.techscience.com/doi/10.32604/or.2026.085665/s1.
