# Supplementary material for: The Impact of Combretastatin A-4 on Cancer Cells and Circulating Tumor Cells (CTCs): A Multi-Assay Approach
Source: Oncol Res. 2026 Sep 14;34(10):1. doi: 10.32604/or.2026.085665 (PMC13591330; doi:10.32604/or.2026.085665)
Supplement: Supplementary file 1 [file OncolRes-34-85665-s001.zip › TSP_OR_85665-Supplementary_Materials.docx]

**Supplementary Material**

**The impact of Combretastatin A-4 on Cancer cells and Circulating Tumor Cells (CTCs): A multi-assay approach**

**Supplementary Section S1**

**Supplementary data for the synthesis of Combretastatin A-4 (CA-4)**

CA-4 was synthesized from commercially available starting material according to a literature procedure [1-3]. The reactions involved are described in the following figure (Supplementary Fig. S1).

**Supplementary Fig. S1.** Synthetic route for the preparation of CA-4.

**Experimental Part**

**General considerations**

All reactants and reagents were obtained from commercially available sources and used without further purification. Unless otherwise specified, key starting materials (including 3,4,5-trimethoxybenzyl alcohol, isovanillin, triphenylphosphine hydrobromide, *tert*-butyldimethylsilyl chloride, and *n*-butyllithium solution 2.5 M in hexanes) were purchased from Sigma-Aldrich/Merck (Darmstadt, Germany) and analytical grade solvents were purchased from ACROS Organics (Geel, Belgium). The nuclear magnetic resonance (NMR) spectra were recorded on a Bruker Avance III HD at 600.13 MHz for the ^1^H-NMR and 150.90 MHz for the ^13^C-NMR, using CDCl_3_ and TMS as an internal standard.

***i) Synthesis of the phosphonium salt of 3,4,5-trimethoxy benzyl alcohol***

To a stirred solution of 3,4,5-trimethoxybenzyl alcohol (446 mg, 2.25 mmol) in a solvent mixture of MeCN (2.5 mL), THF (2.5 mL) and PPh_3_•HBr (1.54 g, 4.50 mmol) was added. Then the reaction mixture was heated at 80°C until completion of the reaction (approx. 2 hours) which is confirmed by TLC analysis using as eluant PhMe/AcOEt 9:1. The mixture was then concentrated under vacuum to a minimal volume, and Et_2_O was added. A white solid precipitate was formed, and was separated by filtration under vacuum. The corresponding phosphonium salt was washed on the filter with cold Et_2_O and was obtained pure as a white solid without further purification.

**NMR spectra:**

**^1^H NMR (600 MHz, CDCl_3_): *δ*** 7.78 (dd, *J=*14.5, 9.7 Hz, 3H), 7.72 (dd, *J=*12.9, 7.8 Hz, 6H), 7.66 (td, *J=*7.7, 3.4 Hz, 2H), 7.61 (td, *J=*7.6, 3.3 Hz, 4H), 6.44 (d, *J=*2.2 Hz, 1H), 5.31 (d, *J=*14.0 Hz, 1H), 3.48 (s, 6H), 3.74 (s, 3H) ppm;

**^13^C NMR (151 MHz, CDCl_3_): *δ*** 153.14, 153.12, 135.06, 135.00, 134.98, 134.74, 134.67, 133.82, 133.76, 130.62, 130.54, 130.19, 130.11, 122.49, 118.21, 117.65, 108.98, 60.95, 60.16, 56.33, 30.91 ppm.

**ii) Synthesis of** ***O*-TBDMS isovanillin**

To a stirred solution of isovanillin (700 mg, 4.60 mmol) in anhydrous DMF (6 mL) was added Et_3_N (1.28 mL, 9.20 mmol) and the resulting reaction mixture was placed in an ice bath (0°C). Then TBDMS-Cl (1.04 g, 6.90 mmol) was added in portions and after the end of additions the reaction was allowed to proceed at room temperature for 2 hours, after which its completion was confirmed by TLC analysis using as eluant PhMe/AcOEt 95:5.

The reaction mixture was subsequently added to cold H_2_O (100 mL) and extracted twice with DCE. The organic phases were collected separately, combined, and washed three times with saturated aqueous NaCl solution. The organic phase was dried over anhydrous Na_2_SO_4_, filtered, and concentrated under vacuum to dryness. A dark orange oily residue was obtained, which was subjected to FCC using an elution system of PhMe/AcOEt 95:5, leading to the isolation of the pure TBDMS-protected derivative as a yellow, low-viscosity oil.

**MS (m/z) (ESI, ES^+^):** C_14_H_22_O_3_Si [M+H]: 267.14

**R**f **(PhMe/AcOEt 95:5):** 0.50

**NMR spectra:**

**^1^H NMR (600 MHz, CDCl_3_): *δ*** 9.81 (d, *J*=2.6 Hz, 1H), 7.48-7.45 (m, 1H), 7.36 (d, J=2.0Hz, 1H), 6.95 (dd, *J*=8.3, 2.5 Hz, 1H), 3.88 (s, 3H), 1.00 (s, 9H), 0.16 (s, 6H) ppm;

**^13^C NMR (151 MHz, CDCl_3_): *δ*** 191.00, 156.72, 145.69, 130.34, 126.39, 120.17, 111.30, 55.68, 25.75, 18.53, -4.53 ppm

***iii) Synthesis of (E)- and*** ***(Z)-tert-butyl(2-methoxy-5-(3,4,5-trimethoxystyryl)phenoxy)dimethylsilanes via a Wittig reaction***

The phosphonium salt of 3,4,5-trimethoxy benzyl alcohol (300 mg, 0.573 mmol) was dissolved in anhydrous THF (9 mL) and stirred under inert conditions for 2 hours. The reaction mixture was placed in an MeCN/liq. N_2_ bath to lower the temperature to -23°C, and then, a solution of *n*-BuLi in hexanes (2.5 M) (1.25 mL, 13.06 mmol) was added dropwise while maintaining the same temperature for 1 hour. Then, the TBDMS-protected isovanillin (153 mg, 0.573 mmol) was added dropwise, and the reaction was allowed to proceed at the same temperature for 4 hours. Subsequently, it was left overnight at room temperature, and its completion was confirmed the following day by TLC analysis using as eluant hexane/AcOEt 9:1.

The reaction mixture was then cooled to 0°C, and cold H_2_O was added under stirring. The organic phase was extracted, and the aqueous phase was washed three times with Et_2_O. The resulting organic phases were collected separately, combined, and washed three times with cold H_2_O. Then, they were dried using anhydrous Na_2_SO_4_, filtered, and concentrated under vacuum to dryness. The resulting yellow oil was purified by FCC using an elution system of hexane/AcOEt 9.5/0.5, yielding the pure *cis*-stilbene as a pale yellow oil and the pure *trans*-stilbene as a white solid, in ratio of 1:1.6. After the purification, the desired *cis*-stilbene was isolated in a 40% yield [2].

**MS (m/z) (ESI-ES^+^):** C_24_H_34_O_5_Si [M+H]: 431.39, [M+Na]: 453.38, [M+K]: 469.32, [2M+Na]: 883.98

**R**f ***cis*-stillbene (hexane/AcOEt 95:5):** 0.17

**R**f ***trans*-stillbene (hexane/AcOEt 95:5):** 0.14

**NMR spectra of the *cis*-stillbene:**

**^1^H NMR (600 MHz, CDCl_3_): *δ*** 6.85 (dd, *J*=8.3, 2.0 Hz, 1H), 6.79 (d, *J*=2.0Hz, 1H), 6.73 (d, *J*=8.3 Hz, 1H), 6.50 (s, 2H), 6.47 (d, *J*=12.1 Hz, 1H), 6.42 (d, *J*=12.1Hz), 3.83 (s, 3H), 3.78 (s, 3H), 3.70 (s, 6H), 0.92 (s, 9H), 0.06 (s, 6H) ppm;

**^13^C NMR (151 MHz, CDCl_3_): *δ*** 153.11, 150.44, 137.11, 133.24, 130.21, 129.84, 128.91, 122.93, 122.42, 111.81, 106.04, 61.02, 56.04, 55.67, 25.77, 18.49, -4.64 ppm

**NMR spectra of the *trans*-stillbene:**

**^1^H NMR (600 MHz, CDCl_3_): *δ*** 7.05 (dd, *J*=8.3, 2.0 Hz, 1H), 7.03 (d, *J*=2.0 Hz), 6.90 (d, *J*=12.1 Hz), 6.84 (dd, *J*=10.3, 2.0 Hz), 6.71 (s, 2H), 3.91 (s, 6H), 3.82 (s, 3H), 3.81 (s, 3H), 1.02 (s, 9H) ppm;

**^13^C NMR (151 MHz, CDCl_3_): *δ*** 153.51, 151.03, 145.31, 137.77, 133.59, 130.57, 128.07, 126.73, 120.64, 118.76, 112.16, 103.48, 71.50, 63.85, 61.10, 56.26, 55.23, 42.74, 31.72, 25.90, 18.62, 14.25, 1.15, -4.45 ppm

***iv) Deprotection of the TBDMS-protecting group***

A solution of the compound of *cis*-stillbene (83.0 mg, 0.1927 mmol) in anhydrous MeOH (0.92 mL) was stirred under inert conditions, and a solution of HCl in dioxane (250 μL) was added. The reaction mixture was heated at 50°C for 2 hours, after which the completion of the reaction was confirmed by TLC analysis using as eluant hexane/AcOEt 3:1.

The reaction mixture was concentrated under vacuum to dryness and then H_2_O and DCM were added. The aqueous phase was extracted twice with DCM. The organic phase was collected, dried over anhydrous Na_2_SO_4_, filtered, and concentrated under vacuum to dryness [1].

The crude colorless oil was purified by FCC using an elution system of hexane/AcOEt 3:1, yielding the pure product, CA-4, as a white solid.

**Melting point:** 115^o^C

**R**f **(hexane/AcOEt 3:1):** 0.23

**MS (m/z) (ESI, ES^+^):** C_18_H_20_O_5_ [Μ+Η]: 431.22, [Μ+Na]: 453.29, [M+K]: 469.25

**NMR spectra:**

**^1^H NMR (600 MHz, CDCl_3_): *δ*** 6.92 (d, *J*=1.7Hz, 1H), 6.79 (dd, *J*=8.3, 1.5 Hz, 1H), 6.73 (d, *J*=8.3 Hz, 1H), 6.52 (s, 2H), 6.47 (d, *J*=12.1 Hz, 1H), 6.41 (d, *J*=12.1 Hz, 1H), 5.53 (s, 1H), 3.86 (s, 3H), 3.84 (s, 3H), 3.70 (s, 6H) ppm;

**^13^C NMR (151 MHz, CDCl_3_): *δ*** 152.99, 145.89, 145.36, 137.28, 132.83, 130.76, 129.62, 129.16, 121.24, 115.17, 110.45, 106.19, 61.05, 56.08, 56.05, 43.58 ppm

**HPLC reverse phase:** C-18 column, length 125mm x 4.6 mm ID, flow rate: 1 mL/min.

Gradient elusion from 50-50 (v/v) ACN/H_2_O to 100 (v/v) ACN over a period of 35 minutes.

The solid was dissolved in 1 mL MeOH

Retention time: 4.5 min.

**Supplementary Fig. S2.** HPLC spectrum of CA-4.

The HPLC spectrum is in accordance with the data provided in the literature and the purity of the compound (CA-4) is ca 99% [2].

**Supplementary Table S1.** Key Characterization Data for Combretastatin A-4 (CA-4).

| Analysis Type | Details & Results |
| --- | --- |
| Appearance | White solid |
| Melting Point | 115 ^o^C |
| Purity (HPLC) | 99% (Retention time: 4.5 min) |
| Mass Spectrometry (ESI, ES+) | C_18_H_20_O_5_ [Μ+Η]: 431.22, [Μ+Na]: 453.29, [M+K]: 469.25 |
| ^1^H NMR (600 MHz, CDCl_3_) | ***δ*** 6.92 (d, *J*=1.7Hz, 1H), 6.79 (dd, *J*=8.3, 1.5 Hz, 1H), 6.73 (d, *J*=8.3 Hz, 1H), 6.52 (s, 2H), 6.47 (d, *J*=12.1 Hz, 1H), 6.41 (d, *J*=12.1 Hz, 1H), 5.53 (s, 1H), 3.86 (s, 3H), 3.84 (s, 3H), 3.70 (s, 6H) ppm |
| ^13^C NMR (151 MHz, CDCl_3_) | ***δ*** 152.99, 145.89, 145.36, 137.28, 132.83, 130.76, 129.62, 129.16, 121.24, 115.17, 110.45, 106.19, 61.05, 56.08, 56.05, 43.58 ppm |

**Supplementary figures of** **NMR spectra**

**Supplementary Fig. S3.** ^1^H NMR (600 MHz, CDCl_3_) spectrum of the phosphonium salt of 3,4,5-trimethoxy benzyl alcohol.

**Supplementary Fig. S4.** ^13^C NMR (151 MHz, CDCl_3_) spectrum of the phosphonium salt of 3,4,5-trimethoxy benzyl alcohol.

**Supplementary Fig. S5.** ^1^H NMR (600 MHz, CDCl_3_) spectrum of *O*-TBDMS isovanillin.

**Supplementary Fig. S6.** ^13^C NMR (151 MHz, CDCl_3_) spectrum of *O*-TBDMS isovanillin.

**Supplementary Fig. S7.** ^1^H NMR (600 MHz, CDCl_3_) spectrum of (*Z*)-tert-butyl(2-methoxy-5-(3,4,5-trimethoxystyryl)phenoxy)dimethylsilane.

**Supplementary Fig. S8.** ^13^C NMR (151 MHz, CDCl_3_) spectrum of (*Z*)-tert-butyl(2-methoxy-5-(3,4,5-trimethoxystyryl)phenoxy)dimethylsilane.

**Supplementary Fig. S9.** ^1^H NMR (600 MHz, CDCl_3_) spectrum of CA-4.

**Supplementary Fig. S10.** ^13^C NMR (151 MHz, CDCl_3_) spectrum of CA-4.

**Supplementary Fig. S11.** Determination of the IC_50_ value of Combretastatin A-4 (CA-4) in H1299 cells after 24 h of treatment. H1299 cells were treated with increasing concentrations of CA-4 for 24 h, and cell viability was assessed by the MTT assay. Viability values were normalized to DMSO-treated control cells (set at 100%). Data are presented as mean ± SD from three independent experiments. The concentration–response curve was generated using nonlinear regression analysis, and the estimated IC₅₀ value was calculated as 10.38 μM. Dotted lines indicate the 50% viability level and the corresponding IC₅₀ concentration.

**Supplementary Fig. S12.** Representative optical microscope images showing colony formation in H1299 (A), HT-29 (B), MDA-MB-231 (C), and CTC-MCC-41 (D) cells under control conditions and following Combretastatin A-4 (CA-4) treatment (10 µM) for 24 and 48 hours. Images captured at 20× magnification. Scale bars represent 10 μm.

**Supplementary Fig. S13.** Representative grayscale images from migration assays showing H1299 (A), HT-29 (B), MDA-MB-231 (C), and CTC-MCC-41 (D) cells under control conditions and after Combretastatin A-4 (CA-4) treatment (10 µM) for 24 and 48 hours. Images were acquired using an optical microscope. Scale bars represent 10 μm.

**Supplementary Fig. S14.** Quantification of microtentacle (McTN)-positive cells in H1299, HT-29, MDA-MB-231, and CTC-MCC-41 models. Cells were treated with Combretastatin A-4 (CA-4) (10 μM) for 1 h and assessed for the presence of McTNs using the TetherChip platform. Data are expressed as the percentage of McTN-positive cells relative to control. McTN analysis was performed using three technical replicate wells per condition, and values represent the combined counts obtained across replicate wells.

**Supplementary Table S2.** Total number of McTN-positive cells identified across three technical replicate wells per condition, under control conditions and after Combretastatin A-4 (CA-4) treatment (10 μM, 1 h) for each cancer cell line.

| Cell line | Control | CA-4 10 μΜ 1h |
| --- | --- | --- |
| H1299 | 21 | 3 |
| HT-29 | 9 | 1 |
| MDA-MB-231 | 18 | 2 |
| CTC-MCC-41 | 35 | 11 |

**References**

**[1]** Huang X, Huang R, Gou S, Wang Z, Liao Z, Wang H. Combretastatin A-4 analogue: A dual-targeting and tubulin inhibitor containing antitumor Pt(IV) moiety with a unique mode of action. *Bioconjug Chem*. 2016;27(9):2132–2148. https://doi.org/10.1021/acs.bioconjchem.6b00353
**[2]** Pettit GR, Singh SB, Hamel E, Lin CM, Alberts DS, Garcia-Kendall D. Isolation and structure of the strong cell growth and tubulin inhibitor combretastatin A-4. *Experientia*. 1989;45(2):209–211. https://doi.org/10.1007/BF01954881

**[3]** Pandey H, Pandey P, Singh S, Negi AS, Banerjee S. Unveiling the future reservoir of anti-cancer molecule—combretastatin A4 from callus and cell aggregate suspension culture of flame creeper (Combretum microphyllum): growth, exudation and elicitation studies. Plant Cell Tissue Organ Cult. 2020; 143:681–691. https://doi.org/10.1007/s11240-020-01951-x
